# A Principal Component of Quality-of-Life Measures Is Associated with Survival: Validation in a Prospective Cohort of Lung Cancer Patients Treated with Stereotactic Body Radiation Therapy

**DOI:** 10.3390/cancers13184542

**Published:** 2021-09-10

**Authors:** Mark K. Farrugia, Han Yu, Gregory M. Videtic, Kevin L. Stephans, Sung Jun Ma, Adrienne Groman, Jeffrey A. Bogart, Jorge A. Gomez-Suescun, Anurag K. Singh

**Affiliations:** 1 Department of Radiation Medicine, Roswell Park Comprehensive Cancer Center, Buffalo, NY 14203, USA; mark.farrugia@roswellpark.org (M.K.F.); sungjun.ma@roswellpark.org (S.J.M.); jorge.gomez@roswellpark.org (J.A.G.-S.); 2 Department of Biostatistics and Bioinformatics, Roswell Park Comprehensive Cancer Center, Buffalo, NY 14203, USA; Han.Yu@RoswellPark.org (H.Y.); adrienne.groman@roswellpark.org (A.G.); 3 Department of Radiation Oncology, Cleveland Clinic Foundation, Taussig Cancer Institute, Cleveland, OH 44195, USA; videtig@ccf.org (G.M.V.); stephak@ccf.org (K.L.S.); 4 Department of Radiation Oncology, State University of New York Upstate Medical University, Syracuse, NY 13210, USA; bogartj@upstate.edu

**Keywords:** lung cancer, NSCLC, SBRT, quality of life, principal component analysis, survival

## Abstract

**Simple Summary:**

There is a paucity of literature on the association between health-related quality-of-life (HRQOL) measures and survival outcomes among patients with early-stage non-small-cell lung cancer following stereotactic body radiation therapy (SBRT). To address this knowledge gap, we performed a secondary analysis of a prospective randomized clinical trial using principal component analysis (PCA). A total of 70 patients were enrolled and completed HRQOL questionnaires prior to and 3 months after SBRT. Using PCA, one of the eigenvectors, PC1, incorporated changes in global health status, functional HRQOL performance, and symptom burden, and it was associated with progression-free survival and overall survival outcomes. Changes in HRQOL measures based on PCA may help identify a subgroup of high-risk patients, and further studies would be warranted to tailor potential additional interventions in this subgroup to improve their outcomes.

**Abstract:**

The association between HRQOL metrics and survival has not been studied in early stage non-small-cell lung cancer (NSCLC) patients undergoing SBRT. The cohort was derived via a post-hoc analysis of a prospective randomized clinical trial examining definitive SBRT for peripheral, early-stage NSCLC with a single or multi-fraction regimen. Patients completed HRQOL questionnaires prior to and 3 months after treatment. Using principal component analysis (PCA), changes in each HRQOL scale following treatment were reduced to two eigenvectors, PC1 and PC2. Cox regression was employed to analyze associations with survival-based endpoints. A total of 70 patients (median age 75.6 years; median follow-up 41.1 months) were studied. HRQOL and symptom comparisons at baseline and 3 months were vastly unchanged except for improved coughing (*p* = 0.02) and pain in the chest at 3 months (*p* = 0.033). PC1 and PC2 explained 21% and 9% of variance, respectively. When adjusting for covariates, PC1 was significantly correlated with progression-free (PFS) (HR = 0.78, 95% CI 0.67–0.92, *p* = 0.003) and overall survival (OS) (HR = 0.76, 95% CI 0.46, *p* = 0.041). Changes in global health status, functional HRQOL performance, and/or symptom burden as described by PC1 values are significantly associated with PFS and OS. The PC1 quartile may facilitate the identification of at-risk patients for additional interventions.

## 1. Introduction

Recently, our group has shown that the post-treatment recovery of health-related quality-of-life (HRQOL) domains is associated with overall survival (OS) in head and neck cancer. To account for the multicollinearity of HRQOL measures, we employed principal component analysis (PCA) [1]. In looking for opportunities to test these findings, we noted that HRQOL measures have been shown to correlate with survival in a number of disease sites, including lung cancer [2,3,4,5,6,7].

Lung cancer is the leading cause of cancer-related death worldwide with 1.8 million deaths from an estimated 2.2 million new cases in 2020 [8]. HRQOL measures have been shown to correlate with survival in a number of disease sites [2,3,4,5,6,7]. However, studies are heterogenous with regard to stage, treatment, and histology, and many are limited to baseline HRQOL information [2,3,4,5,6,7].

Approximately 10–15% of patients present with early-stage non-small-cell lung cancer (NSCLC) [9]. For patients with medical co-morbidities precluding surgical resection, lung stereotactic body radiation therapy (SBRT) has emerged as the standard of care given its excellent outcomes and minimal treatment related toxicity [10,11,12,13,14,15]. However, despite favorable toxicity profiles, up to 30% of patients experienced clinically meaningful deterioration in global and physical HRQOL measures, which persisted up to a year after SBRT [16]. While a number of factors may influence outcomes following SBRT, the impact of health-related quality-of-life parameters has yet to be studied.

To address this knowledge gap and validate the PCA methodology, we analyzed data from a multi-institutional phase II trial of early-stage, peripheral NSCLC randomized to single or three-fraction SBRT [13].

## 2. Materials and Methods

### 2.1. Patient Population

This study cohort was derived from a completed and reported multi-institution, randomized, phase II clinical trial examining one fraction versus three fraction lung SBRT for peripheral stage I–II NSCLC [13]. Institutional Review Board approval from each participating institution was obtained and data was retained at Roswell Park Comprehensive Cancer Center (I-124407) (ClinicalTrials.gov NCT00843726). Of the 98 available patients, 72 had completed baseline and post-treatment HRQOL questionnaires available for analysis. Following analyses (see statistical analysis), 2 patients were excluded as outliers for a total of 70 patients.

### 2.2. Patient Characteristics

The study inclusion criteria have been previously discussed in detail [13]. Briefly, patients with peripheral early-stage and node-negative histologically confirmed NSCLC, and who were deemed surgically ineligible or refused surgery were included. The definition of peripheral tumors was based on RTOG 0236 criteria [10]. Tumor stages T1–T2 (<5 cm) were allowed with staging per the American Joint Committee on Cancer (AJCC) 6th edition. Performance status was reported using Karnofsky performance status (KPS). Diagnostic work up included computed tomography (CT) and positron emission tomography (PET).

### 2.3. Treatment

CT simulation, planning, and treatment delivery methods were previously described [13]. Patients were randomized to either 30 Gy in 1 fraction or 60 Gy in 3 fractions.

### 2.4. Quality-of-Life Questionnaires

The European Organisation for Research and Treatment of Cancer (EORTC) QLQ-C30 and supplementary lung cancer module QLQ-LC13 were used [17]. Patients completed questionnaires at baseline, 6 weeks, then 3, 6, 9, and 12 months after SBRT. For the purposes of this study, we focused on the measures completed at baseline and 3 months. In less than 5% of patients, scores from the 3 month timepoint were missing and data obtained at 6 months were used. Raw values for each HRQOL and symptom domain were standardized via linear transformation to a 0–100 scale such that higher values indicated better functioning for HRQOL scales but worse symptoms on the symptom scales [18]. To determine change in HRQOL and symptoms after treatment, the converted values for the baseline scores were subtracted from the post-treatment scores.

### 2.5. Statistical Analysis

Comparisons between patient demographics and either PC1 or PC2 were performed with the Kruskal–Wallis Test. Differences in baseline and 3 month post-treatment HRQOL measures were assessed via the Wilcoxon signed-rank test. Local failure was determined by either radiographic imaging (CT or PET) or biopsy. For progression-free survival (PFS), either documented progression or death were termed as an event. For overall survival (OS), death due to any cause was termed an event. The date of treatment to date of earliest event was used to calculate OS and PFS. Univariate Cox regression was used to assess associations between relevant variables and outcome. Variables with *p* < 0.2 on univariate study were incorporated into the multivariate model.

PCA was performed as previously described [1]. PCA facilitated dimension reduction, allowing for linear transformation of the difference in post-treatment and baseline measures for the six HRQOL domains and nineteen-symptom scales into orthogonal components. Values that were outside ±3 standard deviations in the first two PCs were considered outliers and removed from the dataset and PCA was re-applied. Thereby, the undue influence of extreme values on the PCA was minimized. To calculate the respective eigenvectors PC1 and PC2, each variable is normalized as Z-scores and multiplied by the relevant variable coefficient or eigenvalue. The loading matrix containing eigenvalues for the calculation of PC1 and PC2 are provided in Supplementary Table S1.

All *p*-values were two sided. Significance was determined by *p* ≤ 0.05. Statistical analyses were performed using R version 4.0.5 and IBM SPSS Version 26.

## 3. Results

Table 1 lists patient and tumor characteristics. Of these, there was a slight female predominance (54.3%), 70% of patients had a KPS of at least 80, it was a majority white patient population (82.9%), stage IA disease was present in 85.7%, and the most common NSCLC histology was adenocarcinoma. The median follow-up was 41.1 months. HRQOL and symptom comparisons at baseline and 3 months were essentially unchanged except for improved coughing (38.1 ± 24.9 vs. 30.0 ± 23.0, *p* = 0.02) and pain in the chest at 3 months (12.4 ± 22.1 vs. 9.2 ± 17.0, *p* = 0.033) (Table 2).

**Figure 1:**
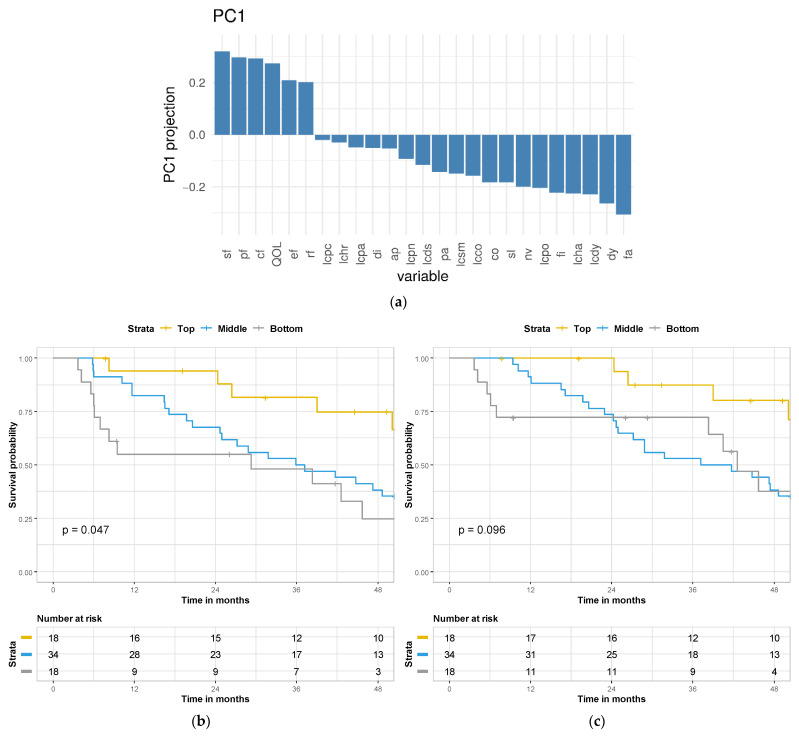
Principal component analysis of the change in health-related quality-of-life (HRQOL) metrics and outcome. (**a**) Waterfall plot demonstrating the weighted contribution of each HRQOL scale. (**b**) Kaplan–Meier analysis of progression-free survival and PC1 quartile. (**c**) Kaplan–Meier analysis of overall survival and PC1 quartile. Definition of HRQOL scales as follows: social functioning (SF); physical functioning (PF); cognitive functioning (CF); global health status (QOL); emotional functioning (EF); role functioning (RF); pain in chest (LCPC); alopecia (LCHR); pain in arm/shoulder (LCPA); diarrhea (DI); appetite (AP); peripheral neuropathy (LCPN); dysphagia (LCDS); pain (PA); sore mouth (LCSM); constipation (CO); insomnia (SL); nausea/vomiting (NV); pain in other parts (LCPO); financial difficulties (FI); hemoptysis (LCHA); dyspnea (LCDY); dyspnea (DY); fatigue (FA).

To evaluate the impact of post-treatment change of HRQOL and symptom scores on outcome, we employed the dimension reduction approach of PCA. PCA demonstrated two principal component (PC) eigenvectors, PC1 and PC2, which explained 21% and 9% of variance, respectively (Supplementary Figure S1). PC1 largely reflects the positive change of the five functional HRQOL domains, global health status, and is contributed in an opposite direction by the change in symptom measures for pain, dyspnea, fatigue, nausea/vomiting, and hemoptysis (Figure 1a) such that higher HRQOL or lower symptom scores at 3 months compared to baseline correlate with a higher PC1 value. The full loading matrix containing the respective eigenvalues for PC1 and PC2, as well the waterfall plot for PC2, can be found in Supplementary Table S1 and Supplementary Figure S1, respectively. Of note, none of the recorded demographic variables (e.g., age, gender, treatment arm) were associated with either PC1 or PC2 (Supplementary Table S2).

In addition to stage (hazard ratio (HR) = 2.4, 95% confidence interval (CI) 1.1–5, *p* = 0.0024), PC1 (HR = 0.83, 94% CI 0.71–0.96, *p* = 0.015) but not PC2 (HR = 0.86, 95% CI 0.7–1.1, *p* = 0.15) was correlated with PFS (Table 3). Only stage was significantly associated with OS (HR = 2.5, 95% CI 1.1–5.5, *p* = 0.024); however, PC1 trended towards significance (HR=0.89, 95% CI 0.77–1, *p* = 0.099) (Table 3). When adjusting for covariates, PC1 was significantly correlated with PFS (HR = 0.78, 95% CI 0.67–0.92, *p* = 0.003) and OS (HR = 0.76, 95% CI 0.46, *p* = 0.041) whereas PC2 was only associated with PFS (HR = 0.78, 95% CI 0.61–0.99, *p* = 0.045) (Table 4).

As PC1 explained a greater percentage of variance of HRQOL measures and was associated with both survival-based endpoints, we focused on PC1 as a prognostic tool. By quartiles, PC1 values can stratify PFS into favorable, intermediate, and poor prognosis groups (*p* = 0.047) (Figure 1b). While similar observations were made when examining PC1 quartile and OS, these findings failed to reach statistical significance (*p* = 0.096) (Figure 1c). 

## 4. Discussion

The current study provided the first evidence that change in HRQOL scores after PCA can be associated with outcomes in early-stage, peripheral NSCLC patients managed with SBRT. Standard HRQOL metrics within the cohort were relatively unchanged at 3 months as compared to baseline, apart from improved coughing (*p* = 0.02) and pain in the chest (*p* = 0.033). However, examination of post-treatment changes in HRQOL revealed powerful prognostic implications. PC1, largely comprised of the change in global health status, the five functional HRQOL domains, and symptoms scales including pain, dyspnea, fatigue, nausea/vomiting, and hemoptysis, was significantly associated with PFS (HR = 0.78, 95% CI 0.67–0.92, *p* = 0.003) and OS (HR = 0.76, 95% CI 0.46, *p* = 0.041) on multivariate analysis. Furthermore, PC1 quartiles successively stratified PFS with similar, albeit non-significant observations with OS highlighting its potential as clinical prognostic factor.

Inclusion of HRQOL endpoints is now emphasized in modern clinical trials and several studies have profiled HRQOL following treatment for lung cancer [19,20]. Within operable NSCLC patients, a review of 19 studies demonstrated decreased physical functioning at 6 months and increased dyspnea and fatigue at 2 years post-treatment [21]. Furthermore, a decline in physical functioning following treatment was observed in NSCLC patients managed with either surgery or SBRT [22]. In stage I NSCLC, surgery was associated with a steeper decline in global health status and physical functioning as compared to SBRT, yet post-treatment reductions in HRQOL were common to either treatment modality [16]. While others have observed declines in HRQOL in NSCLC patients undergoing SBRT, this finding is not universal [23,24]. In a prospective phase II trial, HRQOL measures were stable following SBRT [25]. These findings are supported by the current report and a review of nine studies that also found HRQOL to be relatively unchanged after treatment [26]. Although coughing and pain in chest improved after treatment in current study, the degree of difference was small (<10) and may not be clinically significant [27].

In addition to characterizing how treatment impacts HRQOL in lung cancer, researchers have correlated these metrics with outcome. In a prospective, intention-to-treat study of NSCLC and small cell lung cancer patients undergoing radiotherapy, numerous pretreatment HRQOL measures correlated with mortality with similar findings observed for global health status and physical functioning in another report [5,7]. Decline in physical functioning at 6 months was associated with an increased risk of death in surgically managed NSCLC patients [3]. Moreover, reductions in HRQOL were associated with poor outcome in advanced NSCLC patients undergoing palliative chemotherapy [6].

While median HRQOL values after treatment were stable, PC1 represents the weighted change of select HRQOL scales. Higher PC1, signifying better comparative HRQOL scores at 3 months, was independently associated with prolonged PFS and OS. By quartiles, PC1 demonstrated a grouping of favorable, intermediate, and poor prognosis patients. The mechanism behind these observations is unclear. Patients who experience post-treatment decline may manifest systemic deficits which predispose them to disease recurrence (e.g., immunosuppression) or premature death. At-risk individuals may derive survival benefits from additional interventions such as nursing managements, psychosocial and educational programs, telephone-based interventions for coping skills and support on caregivers, physical activity, and nutritional support [28]. Future work should explore whether addressing changes in HRQOL after treatment for high-risk patients can improve survival-based outcomes.

There are several limitations to this study. First, this is a post-hoc analysis of a prospective clinical trial; consequently, further validation in a large, diverse patient population is necessary. Furthermore, optimal timepoint to assess post-treatment HRQOL changes is not well defined. We suggest that 3 months is adequate to assess decline yet short enough to permit potential meaningful interventions. In addition, HRQOL surveys were performed as optional, and reasons for refusing such surveys were not collected. Patients who refused to participate in HRQOL surveys could have had worse HRQOL measures. Including such high-risk patients could potentially have resulted in more substantial treatment-related changes in HRQOL measures than what our study suggested. Lastly, we recognize that implementation of a PCA-based approach to HRQOL in the clinic is technically more challenging.

PCA, however, offers several advantages when examining HRQOL and outcome. The prognostic value of functional domains and symptoms can vary by disease site [2]. Even within the same disease site, the pertinent HRQOL domains can be discrepant [29]. These discrepancies can be driven by expected differences patient populations, treatments, methodology and, unexpectedly, statistical analysis [2,3,5,6,7,30]. Multivariate Cox regression is a common approach to identify the most relevant HRQOL parameters regarding outcome. However, due to the multicollinearity of functional and symptom domains, this method may erroneously exclude certain scales, thus providing an incomplete portrait regarding HRQOL and outcome [31,32]. An alternative approach is to use cumulative scores, but this provides equal weighting to domains which may truly have no bearing on mortality [33]. Similar to head and neck cancer, the use of PCA for HRQOL measures provided a weighted cumulative score which was informative towards survival-based outcomes further validating this approach [1]. Via this method, we feel these findings can provide a comprehensive and reproducible description of how changes in HRQOL impact the outcome of early-stage NSCLC patients who undergo SBRT.

## 5. Conclusions

HRQOL measures are overwhelmingly stable 3 months following definitive SBRT for early-stage, peripheral NSCLC. However, our study suggests that alterations in global health status, functional HRQOL performance, and/or symptom burden as described by PC1 values are potential prognostic factors for survival outcomes. PC1 quartile may facilitate the identification of at-risk patients for additional interventions.

## Figures and Tables

**Table 1:** Patient demographics.

Age (Years)		Median (IQR)	*n*	%
		75.6 (70.4–81.9)		
Gender	Male		32	45.7%
	Female		38	54.3%
KPS	60		5	7.1%
	70		13	18.6%
	80		28	40.0%
	90		19	27.1%
	100		2	2.9%
	Not reported		3	4.3%
Race	White		58	82.9%
	Non-white		12	17.1%
Histology	Adenocarcinoma		36	51.4%
	SCC		25	35.7%
	NOS		9	12.9%
Stage	IA		60	85.7%
	IB		10	14.3%
Arm	30Gy in 1 fraction		35	50.0%
	60Gy in 3 fractions		35	50.0%
Follow-up (months)		41.1 (22.9–58.0)		

Interquartile range (IQR); Karnofsky performance status (KPS); squamous cell carcinoma (SCC), not otherwise specified (NOS).

**Table 2:** Health-related quality-of-life measures.

Variables	Baseline	3 Months	*p*-Value
	Mean ± st.dev	Mean ± st.dev	
**Global Health Status**			
Global health status	66.0 ± 21.8	66.0 ± 19.5	0.827
**Functional Scales**			
Physical	69.4 ± 20.2	67.2 ± 21.3	0.288
Role	75.1 ± 25.7	72.6 ± 25.7	0.91
Emotional	79.3 ± 21.2	79.2 ± 20.9	0.63
Cognitive	81.7 ± 20.3	84.5 ± 18.9	0.132
Social	80.5 ± 23.7	82.1 ± 22.9	0.498
**Symptom Scales**			
Fatigue	31.8 ± 23.9	34.1 ± 23.4	0.821
Nausea/Vomiting	4.5 ± 9.8	3.8 ± 9.1	0.191
Pain	22.1 ± 26.9	21.4 ± 25.4	0.41
Dyspnea	39.1 ± 32.1	42.0 ± 32.7	0.244
Insomnia	27.6 ± 28.4	21.4 ± 25.4	0.133
Appetite	17.1 ± 23.2	14.8 ± 23.2	0.5
Constipation	10.5 ± 17.5	15.0 ± 27.1	0.281
Diarrhea	10.5 ± 19.3	8.1 ± 20.8	0.233
Financial Difficulties	17.1 ± 28.8	15.2 ± 25.2	0.401
**LC13 Symptom Scales**			
Dyspnea	31.9 ± 25.8	31.0 ± 25.0	0.91
Coughing	38.1 ± 24.9	30.0 ± 23.0	0.02
Hemoptysis	1.9 ± 9.6	1.4 ± 8.9	0.783
Sore Mouth	3.3 ± 11.6	2.4 ± 10.3	0.366
Dysphagia	6.2 ± 16.3	7.6 ± 16.2	0.822
Peripheral neuropathy	16.2 ± 28.2	14.5 ± 26.5	0.777
Alopecia	4.3 ± 11.2	7.1 ± 17.9	0.573
Pain in chest	12.4 ± 22.1	9.2 ± 17.0	0.033
Pain in arm	23.3 ± 32.3	17.1 ± 25.2	0.124
Pain, other	28.1 ± 30.9	24.6 ± 30.6	0.456

Standard deviation (st.dev).

**Table 3:** Univariate Cox regression analysis.

Variables	Progression-Free Survival		Overall Survival	
	HR (95% CI for HR)	*p*-Value	HR (95% CI for HR)	*p*-Value
Age (<75, ≥75)	1.3 (0.72–2.5)	0.35	1.5 (0.76–2.8)	0.25
Gender	0.77 (0.42–1.4)	0.39	0.69 (0.37–1.3)	0.24
KPS	0.99 (0.96–1.02)	0.49	0.98 (0.95–1.01)	0.23
Race (white, non-white)	1.1 (0.51–2.4)	0.79	1.3 (0.6–2.9)	0.49
Stage (IA, IB)	2.4 (1.1–5)	0.024	2.5 (1.1–5.5)	0.024
Arm (30 Gy/1 fx, 60 Gy/3 fx)	1.1 (0.59–2)	0.8	1 (0.55–1.9)	0.93
Histology	0.81 (0.53–1.2)	0.34	0.68 (0.42–1.1)	0.11
PC1	0.83 (0.71–0.96)	0.015	0.89 (0.77–1)	0.099
PC2	0.86 (0.7–1.1)	0.15	0.9 (0.72–1.1)	0.36

Karnofsky performance status (KPS); fraction (fx); principal component (PC); confidence interval (CI); hazard ratio.

**Table 4:** Multivariate Cox regression analysis.

Variables	Progression-Free Survival		Overall Survival	
	HR (95% CI for HR)	*p*-Value	HR (95% CI for HR)	*p*-Value
Stage (IA, IB)	4.0 (1.7–9.1)	<0.001	3.3 (1.4–7.8)	0.007
Histology			0.76 (0.46–1.3)	0.287
PC1	0.78 (0.66–0.92)	0.003	0.85 (0.72–0.99)	0.041
PC2	0.78 (0.61–0.99)	0.045		

Principal component (PC); confidence interval (CI); hazard ratio.

## Data Availability

Farrugia and Singh had full access to all the data in the study and take responsibility for the integrity of the data and the accuracy of the data analysis. The data underlying this article cannot be shared publicly for the privacy of individuals that participated in the study. The data are available from the corresponding authors upon reasonable request.

## Supplementary Materials

The following are available online at https://www.mdpi.com/article/10.3390/cancers13184542/s1, Figure S1: Principal component eigenvectors using dimension reduction approach and the waterfall plot for PC2, Table S1: Loading matrix containing the respective eigenvalues for PC1 and PC2, Table S2: Comparisons between patient demographics and either PC1 or PC2 using the Kruskal–Wallis Test
