# Supplementary material for: A Principal Component of Quality-of-Life Measures Is Associated with Survival: Validation in a Prospective Cohort of Lung Cancer Patients Treated with Stereotactic Body Radiation Therapy
Source: Cancers (Basel). 2021 Sep 10;13(18):4542. doi: 10.3390/cancers13184542 (PMC8469499; doi:10.3390/cancers13184542)
Supplement: Supplementary file 1 [file cancers-13-04542-s001.zip › cancers-1333159-supplementary.pdf]

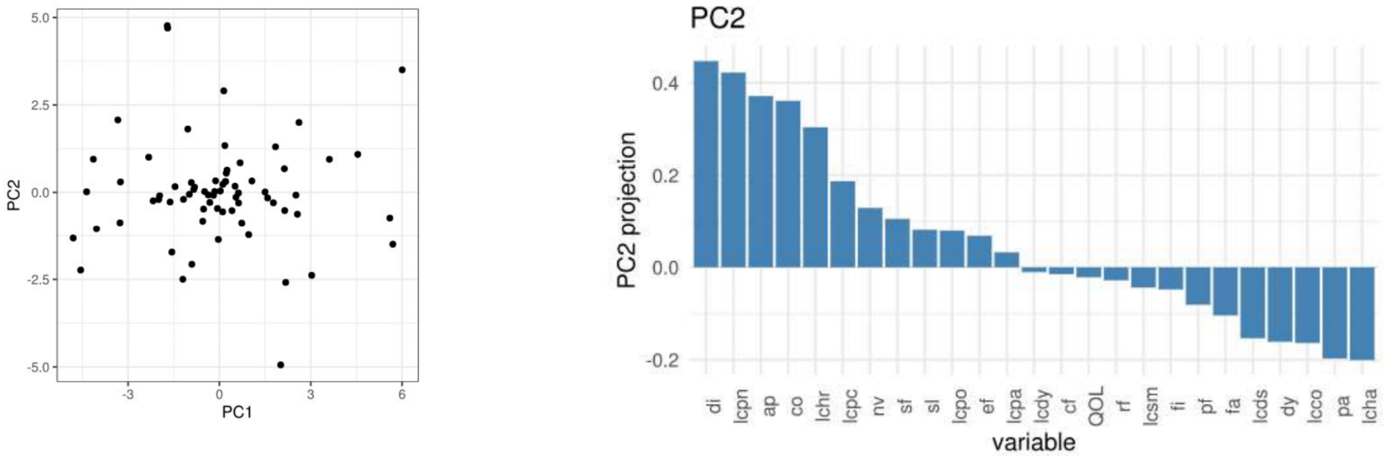

Figure S1. Principal component eigenvectors using dimension reduction approach and the waterfall plot for PC2.

Table S1. Loading matrix containing the respective eigenvalues for PC1 and PC2.

	**PC1**	**PC2**
pf	0.296566	−0.08106
rf	0.201688	−0.02814
ef	0.2096	0.068444
cf	0.293558	−0.01499
sf	0.320354	0.105731
QOL	0.273354	−0.02157
fa	−0.30727	−0.10404
nv	−0.1991	0.129466
pa	−0.14276	−0.19722
dy	−0.264	−0.16111
sl	−0.18298	0.081258
ap	−0.05254	0.371597
co	−0.18281	0.361258
di	−0.05076	0.447343
fi	−0.22275	−0.04803
lcco	−0.15689	−0.16373
lcha	−0.22575	−0.20058
lcsm	−0.14927	−0.04386
lcds	−0.11603	−0.15338
lcpn	−0.09303	0.422428
lchr	−0.03052	0.304172
lcpc	−0.02072	0.187325
lcpa	−0.04908	0.0324
lcpo	−0.20456	0.079528
lcdy	−0.22877	−0.01034

Table S2. Comparisons between patient demographics and either PC1 or PC2 using the Kruskal–Wallis Test.

	**PC1***	**PC2 ***
Age (years) (<75, ≥75)	0.66	0.44
Gender	0.73	0.77
KPS (80+, <80)	0.6	0.25
Race (white, non-white)	0.85	0.27
Stage (IA, IB)	0.28	0.23
Arm (30Gy/1fx, 60Gy/3fx)	0.71	0.91
Histology (Adeno, SCC/NOS)	0.09	0.12

* *p*-value; Karnofsky Performance Status (KPS); Squamous cell carcinoma (SCC), Not otherwise specified (NOS).
